# Personal care expectations: Photovoices of Chinese ageing adults in Hong Kong

**DOI:** 10.1111/hsc.12940

**Published:** 2020-01-09

**Authors:** Xue Bai, Daniel W. L. Lai, Chang Liu

**Affiliations:** ^1^ Department of Applied Social Sciences The Hong Kong Polytechnic University Hong Kong China

**Keywords:** care expectations, frailty, Hong Kong, older Chinese adults, photovoice, retirement

## Abstract

The increasing ageing population contributes to growing demands for personal care that fulfils ageing adults’ preferences and expectations. This study explored Chinese ageing adults’ expected forms and sources of future personal care and the factors influencing care expectations. A qualitative photovoice research method – which integrated photography, interviews and group discussions – was used for data collection between January and April 2016. Through purposive sampling, 36 community‐residing participants aged 51–80 years took photographs that captured personal care preferences or expectations within individual, familial and societal contexts. Participants described feelings of worry, uncertainty and unpreparedness for future care needs and arrangements. They preferred practicing self‐care for as long as possible and remaining in their homes and communities (“ageing in place”) through support from assistive technologies, family members or home‐based and community services. Institutional care was regarded as the last resort. The findings reflected discrepancies between ageing adults’ care preferences and realities and their ambivalent attitudes towards filial care when switching between roles. Confronted with the increasingly unreliable family care, financial resources and insufficient community services, participants anticipated receiving institutional care that would be less satisfying or that they would dislike. As caregivers, ageing adults displayed strong commitment to filial obligations, whereas when perceiving themselves as care receivers they felt that they could not expect care from their children because of practical considerations. By understanding preferred care forms and sources, actors can devote resources, policies and interventions to support self‐care through proactive planning and technological advancement, foster “ageing in place” through family and community care, and improve institutional care to enable ageing with dignity.

**What is known about this topic**

- Changing sociocultural and demographic contexts have altered the meaning and practice of filial obligation and modified ageing adults’ care expectations.
- Older adults’ care preferences are shaped by degree of relation to care providers, and one source of care may be replaced by other sources if necessary.

**What this paper adds**

- Ageing adults experienced feelings of worry, uncertainty and unpreparedness for future care needs and arrangements, discrepancies between care preferences and realities, and ambivalent attitudes towards filial care.
- Self‐care and ageing in place through proactive planning, technological advancement and home‐based services should be fostered. Institutional care quality should be improved to ease ageing adults’ fear of formal care.

## 1 INTRODUCTION

A growing ageing population inevitably results in increased chronic morbidity and functional disability and contributes to increased demands for personal care and assistance. In Hong Kong, more than 70% of adults aged 60 years or older have chronic diseases, 20% experience functional impairment and approximately 25% require assistance with daily living activities (Census & Statistics Department, 2009). In Chinese culture, filial obligation in providing care and “ageing in place” is a common value, and older adults may rely on family members for primary support. However, sociocultural and demographic changes over decades have greatly altered traditional beliefs and practices in Hong Kong.

The elderly people dependency ratio (the number of persons aged 65 or older per 1,000 persons aged between 15 and 64) rose significantly from 142 in 1996 to 218 in 2016 because of declining birth rates over the past two decades (Census & Statistics Department, 2018). The proportion of older adults living with their children has decreased from 53.4% in 2006 to 48.5% in 2016, whereas the proportion of older adults living alone increased from 11.6% to 13.1% in the same time period (Census & Statistics Department, 2018). Aside from demographic changes, evidence also indicates that cultural values in Chinese society are steadily shifting from collectivism towards individualism (Zhu & Ouyang, 2015). Individual rights now seem to receive more attention than do family responsibilities. Studies have reported that the traditional value of filial piety, which demands absolute obedience of children to their parents, is declining in China (Long & Feng, 2007). Because of globalisation and the pursuit of self‐fulfilment, Chinese young people are more likely to leave home to seek employment opportunities. Geographical distance and considerable life and work pressure increase the difficulty experienced by young people in providing financial, emotional and personal care for their parents. Under such circumstances, the meaning and practice of filial obligations have changed, and Chinese ageing adults have modified their care expectations (Bai, 2019; Chou, Kröger, & Pu, 2015; Chow & Bai, 2011; Zhan, Feng, & Luo, 2008). Understanding and respecting ageing adults’ care expectations is crucial in promoting their well‐being in later life (Bai, 2019; Heid et al., 2014).

Care expectations refer to beliefs regarding preferred care forms and sources in anticipation of future assistance across multiple domains (Bai, 2019). Research and theories have emphasised the importance of care planning in preparation of ageing and have focused on care expectations. Proactive coping theory (Aspinwall & Taylor, 1997) emphasises the importance of formulating concrete plans to address future care needs. Care planning promotes communication among ageing adults, family members and professionals (Chan & Pang, 2010; Malcomson & Bisbee, 2009; Musa, Seymour, Narayanasamy, Wada, & Conroy, 2015). By understanding preferred care forms and sources, government actors can enable resources, policies and interventions to enhance older adult care quality.

Personal care expectations comprise various needs, including assistance with daily living activities such as maintaining hygiene, preparing meals, housekeeping, shopping, attending appointments and managing finances (Backman & Hentinen, 1999; Jacobs, Broese van Groenou, Boer, & Deeg, 2014; Lo & Russell, 2007). They also comprise various care sources. Theoretical approaches address the interaction between informal and formal sources of older adult care needs (Bai, 2019; Denton, 1997). The compensatory view proposes that individuals’ care preferences are determined by their relation to care providers (Cantor, 1991; Cantor & Brennan, 2000), and when informal care is unavailable or inadequate, formal care is required to compensate for this lack of informal care (Iecovich, 2014). The complementary view suggests that individuals may prefer multiple forms of care from formal and informal sources with various care responsibilities that are suited for different tasks (Cheung, Kwan, & Ng, 2006; Katz et al., 2003; Litwak, 1985). However, only limited research (e.g. Stoller & Earl, 1983; Wellman & Wortley, 1990) conducted in western societies has lent empirical support for these two approaches. Financial constraints, inadequate health and community care, and sociocultural contexts and values affect the applicability of existing theories to Chinese ageing populations of different sociocultural, economic and demographic context.

The socioeconomic status of older adults in China may differ from that of older adults in Western countries, who are afforded more prevalent and institutionalised social security and retirement protections. Many older adults in Hong Kong do not benefit much from pensions, and old‐age poverty is widespread (Lee, To, & Yu, 2014; Ng, 2016). Hong Kong's current retirement protection system does not provide universal pensions for older people: the retirement scheme of the Mandatory Provident Fund (a mandatory, managed and fully funded contribution scheme to help the ageing workforce save for retirement; Mandatory Provident Fund Schemes Authority, 2017) launched only two decades ago cannot provide sufficient pension payments due to the limited accumulation in personal accounts. The means‐tested social security scheme elicited severe labelling effects for its applicants; and the payments provided by non–means‐tested programmes are insufficient (Bai, 2019). For many older adults in Hong Kong, inferior financial status may limit care possibilities. Moreover, the high population density of Hong Kong, a geographically small city with nearly 8 million people and one of the world's highest longevity rates, can cause overcrowding of services and facilities (Chak, Inclan‐Valadez, Leung, Taylor, & Yip, 2011; Yeh, 2011). Increasing care needs from the large ageing population coincide with the limited space available for comprehensive community‐based care provision, and long waiting times for public services and facilities frustrate older applicants.

Existing theories and previous research findings may not align with the current situation in Hong Kong. Only by examining the care preferences and expectations of ageing adults can service providers and policy‐makers effectively address their challenges and uncertainties.

### 1.1 Research objectives and questions

This study (a) explored ageing adults’ expected types and sources of future personal care and (b) identified factors that influence personal care expectations within changing demographic and sociocultural contexts. This study addresses a key gap in existing research, which is largely centred on Western conceptualisations of care planning, and provides insight into ageing adults’ care preferences, expectations and experiences in Hong Kong. Detailed understanding of personal care expectations, facilitators and barriers can inform appropriate older adult care services in Hong Kong and services in other countries with similar sociocultural backgrounds and demographic and geographical characteristics.

## 2 METHODS

### 2.1 Research design

The data used in this paper are part of a larger study examining the financial, emotional, medical and personal aspects of ageing adults’ care expectations. Several studies on their financial care expectations have been published (Bai, 2019), whereas this study focuses on the personal care aspect by using a qualitative photovoice research design. This participatory research approach, developed by Wang and Burris (1997), integrates photography, interviews and group discussions. Participants took photographs that captured their views on care preferences or expectations within individual, familial and societal contexts and that individually and collectively reflected care expectations, facilitators, barriers and actions to address concerns. Ethical approval was obtained from the Human Subjects Ethics Sub‐Committee at the first author's institution.

### 2.2 Participants and recruitment

Participants were recruited from senior centres in the New Territories, Kowloon and Hong Kong Island and the Hong Kong Polytechnic University's Institute of Active Ageing by using a purposive sampling approach. Invitation letters seeking assistance with recruitment were sent to senior centres. Participants were included who were community‐dwelling, aged 50 or older, spoke Cantonese or Mandarin, and had good visual, motor and cognitive ability that enabled the use of digital cameras or smartphones. Ageing adults spanning a wide age range were recruited to observe potential cohort differences.

### 2.3 Procedures

**Figure 1:**
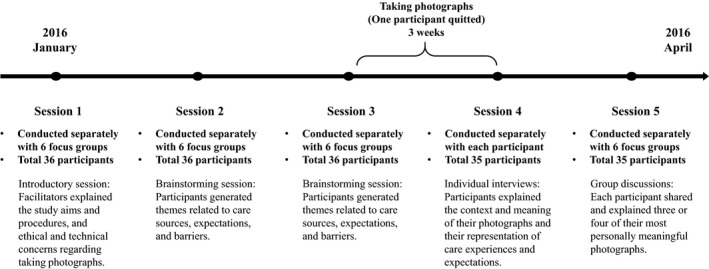
Flow diagram for the research procedures

As shown in Figure 1, the photovoice process (from January to April 2016) was led by two trained researchers and comprised five sessions (Wang & Burris, 1997). Participants were invited to sign consent forms before the first session. Researchers divided all participants into six groups, and all sessions were conducted with each group separately. *Session I* was an introductory session conducted in early January. The facilitator explained the study aims and procedures, and ethical and technical concerns regarding taking photographs with cameras or smartphones and obtaining consent from people in the photographs. *Sessions 2* and *3* were conducted 1 and 2 weeks later and were group brainstorming sessions to generate themes related to care sources, expectations and barriers. Participants were then given 3 weeks to take 10–15 photographs of people, places or experiences representing the themes. At this stage, one participant quit the study for personal reasons. *Session 4* involved semi‐structured individual interviews (approximately 1 hr each), which were conducted in mid‐February after all participants had finished taking photographs. Following an adapted SHOWeD approach (Lewis & Lewis, 2014), participants explained the context and meaning of their photographs and their representation of care experiences and expectations. Details of all questions involved in the SHOWeD approach are available elsewhere (Bai, 2019). *Session 5* involved a group discussion session conducted in early March after all individual interviews were finished. Each participant shared and explained three or four of their most personally meaningful photographs. Upon completion, participants received a HK$100 (roughly US$12.82 at the time of writing) supermarket coupon. During the entire process, if participants had any questions or feedback regarding the research, group facilitators discussed with them and determined whether improvement of session contents was needed. Facilitators wrote reflective notes after each session to record their observations and reflections.

### 2.4 Data analysis and interpretation

All interviews and discussions were audio‐recorded with participants’ consent and transcribed verbatim. Transcripts, photographs, participant demographic data forms and facilitators’ reflection notes were included in the data analysis. This analysis involved three levels of coding: (a) “open coding,” which identified distinct concepts and categories in the transcripts to develop preliminary analysis themes, (b) “axial coding,” which categorised themes and explored the relationships between them, and (c) “selective coding,” which formulated core themes and integrated, refined and related all other themes with the core themes. To ensure interrater reliability, the two researchers separately coded the first group's transcripts and compared codes. The research team collectively determined the coding structure for the remaining transcripts. NVivo 11 was used to assist data management and analysis.

## 3 FINDINGS

In total, 36 participants aged 51–80 years (18 women and 18 men) completed the photovoice process. Of them, 28 were aged 50–69 years (“young‐old”), five were aged 70–79 years (“middle‐old”) and three were aged 80 years or older (“old‐old”). Regarding family status, 20 were married and 16 were single, divorced, separated or widowed. Furthermore, 27 had at least one child, and nine were childless. Regarding living arrangements, 13 lived alone. Five worked full‐time or part‐time and 31 were retired. More details concerning participants’ demographic characteristics are available in a published article (Bai, 2019). In this study, findings concerning participants’ personal care expectations were categorised into four main themes: worry and uncertainty regarding personal care and independence, expectations of support for ageing in place, institutional care as a last resort for extensive care needs and discrepancies and ambivalence in personal care expectations.

### 3.1 Worry and uncertainty regarding personal care and independence

**Figure 2:**
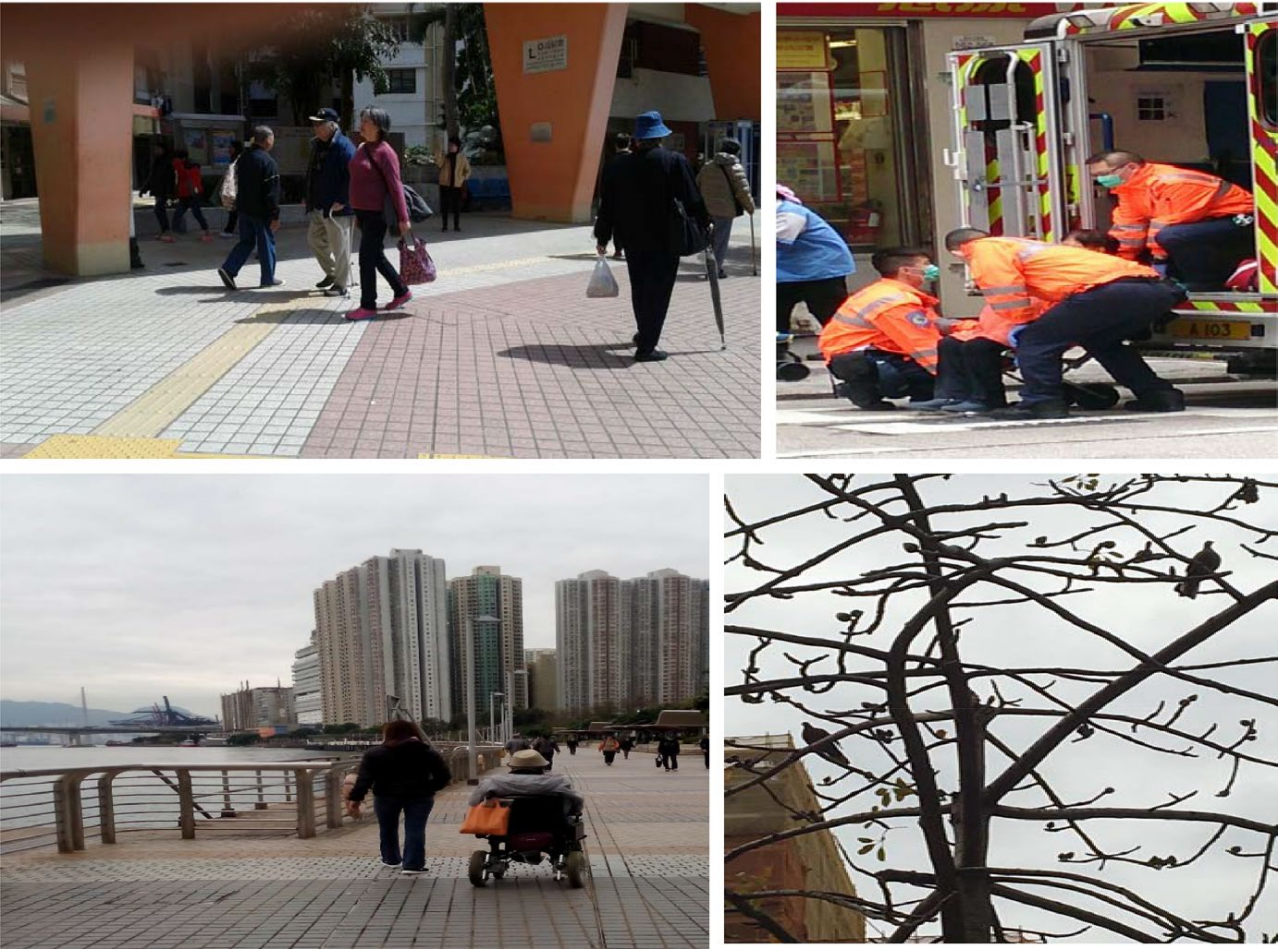
Participant photos illustrating independence and autonomy. Top left: older people walking down in the street. Bottom left: older woman using an electric wheelchair. Top right: an older person taken away by paramedics. Bottom right: a bare tree

Participants’ narratives reflected worries and feelings of uncertainty regarding future care needs and arrangements. Figure 2 illustrates that these included preferences for independence and fears about loss of mobility and autonomy. All age groups expressed similar worries concerning future care needs.

#### 3.1.1 Worries concerning future care needs

Care expectations were based on beliefs and feelings regarding future care needs. Several participants described feelings of worry and fear in this respect, including in terms of physical and emotional aspects, such as loss of mobility, control and independence. Choi (female, age 68) photographed an older person being taken away by paramedics (Figure 2, top right):

When I get old, this will happen to me. I will be incapable of walking on my own and need to be lifted up and down by others. I can see what is going to happen when I become incontinent and lose my mobility. I am very afraid of losing control of myself.

Most participants were afraid of receiving inadequate care when their physical status deteriorates. Because of public media reports, they felt their life might end in tragedy without proper care:

I am quite worried about… my care arrangements. I once lived in Yau Tong, and an older man died without being noticed for a long time. Only when neighbors smelled his dead body did they find out about his death… I am worried that the same thing will happen to me. (Lo, male, age 80)

For others, loneliness was their biggest fear: “I am very afraid of feeling lonely… Many older people face the same problem. They are afraid of losing their company, especially when they lose physical mobility” (Chan, female, age 60).

#### 3.1.2 Feelings of unpreparedness and uncertainty regarding care arrangements

Several participants described feeling unprepared and uncertain regarding personal care expectations and arrangements or avoiding thinking about future needs: “I have never thought about it. Thinking about this problem will give me negative feelings… I made no plans for this… I am not sure if my family will take care of me” (Lau, male, age 70). Fung (male, age 70) showed a photograph of a bare tree (Figure 2, bottom right): “I do not think about [personal care]. I do not know what to do about it. I do not know how I will go through my last few years when I cannot move.”

Participants with favourable financial status or with strong intergenerational relationships generally felt better prepared: “I expect that my son can take care of me. He promised me he will care for me until I die… My son and I have a very good relationship. He's always told me not to worry” (Lau, male, age 66).

#### 3.1.3 Preference for self‐care and independence

Participants of all age groups wished to stay independent and care for themselves for as long as possible. Self‐care was prioritised over other types of care, including family care. Participants valued independence, including making their own decisions, maintaining control over their lives and avoiding being burdensome by depending on others. Au Jeong (female, age 72) presented a photograph of older people walking “freely and independently” (Figure 2, top left): “I try not to cause trouble for others… I don't want to depend on anyone… I feel peaceful and happy when I do not need to count on others.” Chan (female, age 63), photographed an old woman who was using an electric wheelchair (Figure 2, bottom left) to represent autonomy. Many participants wished to engage in self‐care until they were incapable of doing so to avoid seeking assistance unless absolutely necessary:

I don’t like to think about losing independence. Even when I’m not in good health, I will take care of myself. I don’t like to ask for assistance. It’s unfair for other people to take care of you when you are not in a good condition. (Fung, male, age 70)

#### 3.1.4 Strategies for self‐care and independence

**Figure 3:**
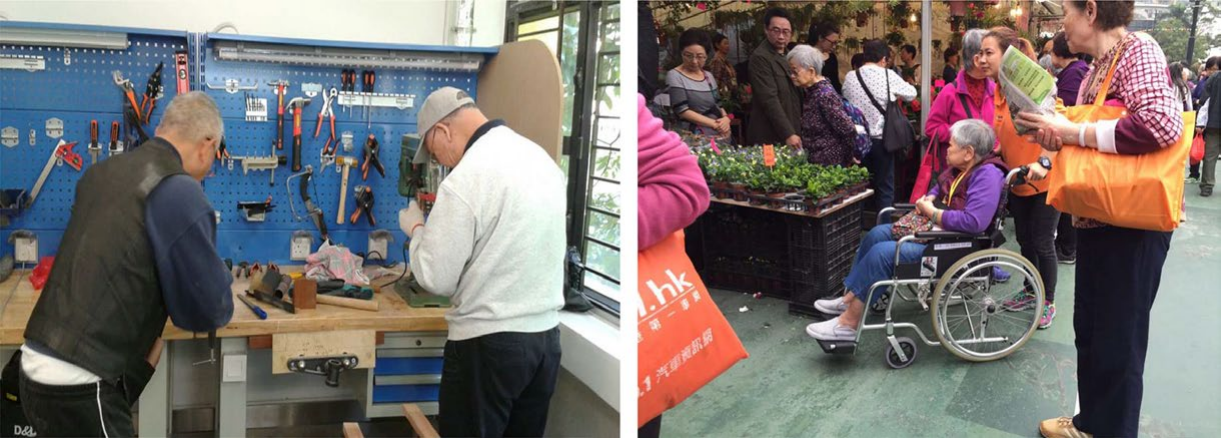
Participant photos illustrating activity participation. Left: older friends in a workshop together. Right: older people participating in outdoor activities

Strategies to maintain independence and well‐being broadly centred on participation in activities such as physical exercise, employment, volunteer work and outdoor activities, as illustrated in Figure 3. Lau (male, age 70) photographed friends working together: “There could be more employment opportunities for older people to make good use of their expertise and work experience.” Lau (male, age 66) photographed older people participating in outdoor activities with support from volunteers: “These volunteers come to help…Hopefully there will be more activities. This would make older people very happy.”

More young‐old participants mentioned new assistive technologies and had greater expectations of innovative caring developments being made to meet their future needs than other age groups. “We may think about how we can develop some automated machines to help us with personal care… We could automate more household products” (Ng, male, age 51). Chan (female, age 63) presented a photo of a woman in an electric wheelchair: “I once saw this older person using an electronic wheelchair. It was quite convenient… [This] is very helpful for older people to gain autonomy.”

### 3.2 Expectations of support for ageing in place

**Figure 4:**
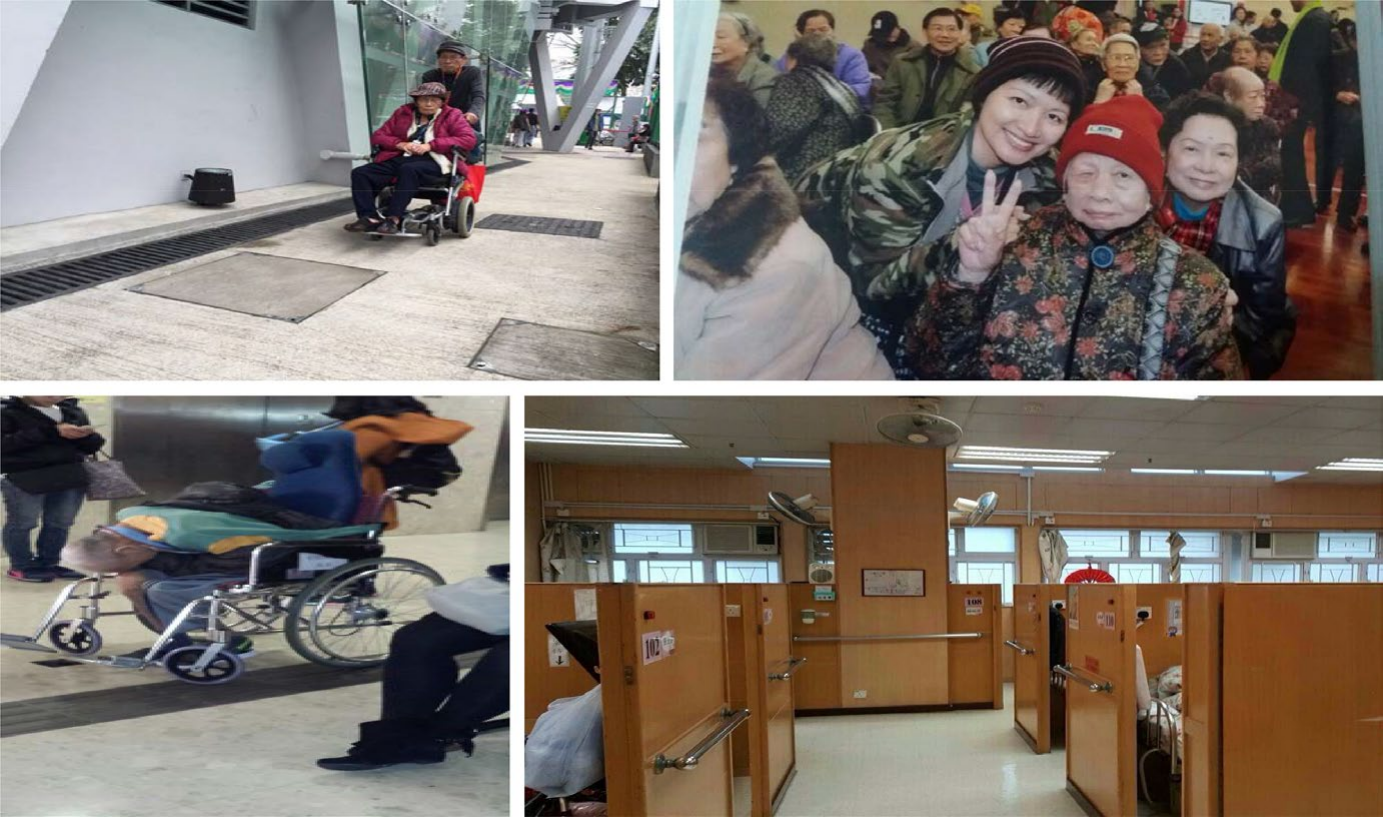
Participant photos illustrating perspectives on home‐based and institutional care. Top left: An older man pushing his wife in a wheelchair. Top right: A participant with an older woman. Bottom left: An older man left in a wheelchair in a nursing home. Bottom right: Inside a government‐subsidized nursing home

When asked about their expectations of care when they become unable to provide self‐care, participants expressed the desire to remain in their homes and communities, known as “ageing in place” (Krothe, 1997). They identified various type of support to meet care needs while living in a familiar social and physical environment and maintaining some independence. Participants’ perspectives on home‐based and institutional care are illustrated in Figure 4.

#### 3.2.1 Family care as the most preferred care form for ageing in place

When older adults lose the ability to provide self‐care, care from family members is preferred over that from formal providers: “Personal care is better when carried out by family members. It requires patience and a loving heart… Nursing home staff only do very superficial things. Volunteers only help for a short time” (Lam, male, age 66). Several expected care from children: “Older people will feel very happy if their children take them out. Younger generations should pay more attention to older people's needs and care more about them” (Man, female, age 66). Others expected care from spouses: “When I am in very poor health, I will choose to live in a nursing home. Before that, I hope that my wife can take care of me at home” (Lai, male, age 59). Lam (male, age 66) showed a picture of a man pushing his wife in a wheelchair (Figure 4, top left):

Most people believe that when a husband gets sick, his wife will take good care of him… This man is caring for his wife… If one day my wife has a stroke, I will accompany her and look after her.

Among all participants, only men expressed expectations of receiving care from a spouse. This is possibly due to conventional labour divisions in the domestic domain. Wives are normally expected to undertake caregiving tasks for the whole family, so women may less often expect their husbands to act as caregivers.

#### 3.2.2 Home‐based and community care services as supplements to family care

Paid home‐based and community care services are crucial care sources for enabling ageing in place. For those with favourable financial status, live‐in or part‐time domestic workers were attractive alternatives to family care:

My father used to disagree with hiring a foreign domestic worker, but now my family cannot live without the worker… She can accompany you when you go to the hospital… They are very helpful. (Ng, male, age 51)

Participants expected the government to provide more home‐based and community care services to meet the care needs of older people:

Community service providers are trained to care for older people, so they know what older people actually need… The services are helpful since some older people do not want a stranger living in their home. (Ling, female, age 60)

When family care is unavailable or insufficient, such services can prolong ageing in place and prevent institutionalisation. Ling presented a photograph of an institutionalised woman (Figure 4, top right):

The government could offer some help for older people’s families to take care of them at home… I once was the caregiver of my grandmother, but I did not have enough money to take care of her… I had to send her to a nursing home.

### 3.3 Institutional care as a last resort for extensive care needs

Participants across age groups appeared reluctant to use institutional care because of the desire to grow old at home, fears regarding living conditions in nursing homes and barriers to accessing preferred facilities. However, many expected to enter nursing homes as a last resort because of extensive care needs: “When I cannot take care of myself, I have to go to a nursing home… I will go there only when I’m dying… I hope I can die before I go there” (Yau, male, age 59).

#### 3.3.1 Fears regarding institutional care

Participants feared institutional care because of concerns regarding poor care quality, staff attitudes and behaviours, and lack of privacy and dignity:

I really cannot trust the staff in nursing homes. It was once reported that older people living in a private nursing home were left naked on an open‐air podium for a long time before staff took them to shower. They have no privacy and dignity. (Lau, male, age 66)

Participants mentioned public criticism and media portrayals of poor conditions and maltreatment of residents in private institutions. Han (female, age 60) angrily described a photograph of an older man left in a wheelchair (Figure 4, bottom left):

This was in a nursing home. This older man may be too weak to sit on the wheelchair. The staff didn’t dress him well and just left him there. How could they treat him like that?… I really hope this won’t happen to me… Totally no dignity.

Participants also described boredom, a lack of privacy and dependence on others:

Many older people who are living in nursing homes do not want to stay alive anymore… life there is too boring. They totally lose interest in life… They only wait for eating meals, sleeping, and going to the washroom. Every day is the same. (Lau, male, age 66)

Participants described wishes or expectations for nursing homes: “If every person could have a private space… and have more activities to take part in, they would not have too many negative thoughts about nursing homes” (Chan, female, age 60). Lau presented a photograph of volunteers taking older people outdoors: “Nursing homes should organize more activities to enrich older people's lives… Older people would then be very happy.”

#### 3.3.2 Preferences for subsidised residential care

Participants described government‐subsidised nursing homes as more affordable than private ones, with better care quality and living environments. Chan (female, age 60) presented a photograph of a government‐subsidised home (Figure 4, bottom right): “The living conditions are quite good… Families don't need to pay much in service fees… We all hope to live in a government‐subsidized nursing home.” These homes had more technological devices to assist with care delivery: “This is a disabled older man. This facility could automatically lift him up… It's much safer and more comfortable… It would be great if I could live here” (Wong, male, age 64). However, waiting times for government‐subsidised facilities prevent many people from perceiving this as feasible: “Older people have to wait for a very long time to enter a public nursing home… When they are accepted, they are almost dead” (Hoi, female, age 66).

### 3.4 Discrepancies and ambivalence in personal care expectations

When participants described preferences and expectations for personal care, their narratives revealed discrepancies between care preferences and realities and their ambivalent attitudes towards filial care. Such discrepancies and ambivalence were more frequently mentioned by young‐old participants than by older age groups, possibly because they were at a life stage of both providing care for their own parents and envisioning their future needs, and this cohort was more influenced by changing cultural values and practical limitations. Their care expectations were characterised by these new features.

#### 3.4.1 Discrepancies between care preferences and reality

Discrepancies existed between care preferences and real situations, which were limited by unreliable family care, financial resources and insufficient community services. Several participants felt that expecting care from family and domestic helpers may be unrealistic and thus only expected institutional care. Mo (female, age 66) presented a picture of two women walking down the street:

A domestic helper is accompanying this older woman… This older woman can live in her own home at this age… I hope I can be like her… but I do not have the money to hire a domestic helper… If I need care someday, I will have to go to a nursing home.

Confronted with the reality that their children may be unable or unwilling to provide future care, participants anticipated receiving institutional care that would be less satisfying or that they would dislike:

If one day I become too old to take care of myself, I think that my children may not be able to take care of me… They have no time and no money… I cannot expect anything but living in a nursing home. I have to accept this. (Hoi, female, age 66)

#### 3.4.2 Ambivalent attitudes towards filial care

Participants had clear ideas regarding the advantages and disadvantages of care options. When asked about care plans for parents, most participants considered filial care to be the best choice: “If my mom's physical condition becomes poor, I will look after her until she dies… It is a promise… She prefers to live at home rather than in a nursing home” (Lai, male, age 59). However, when considering themselves to be care recipients, participants did not take filial care for granted and were unwilling to create burdens for their children, even though they maintained the idea of ageing at home as the ideal scenario:

I accept living in a nursing home myself… When I become frail, I cannot count on my daughter to take care of me at home… I will not let my mom go to nursing home. The care quality is very poor there. (Hoi, female, age 66)

Such ambivalent attitudes towards filial care reflect the discrepancy between care preferences and reality.

## 4 DISCUSSION

### 4.1 Conceptualising personal care expectations

This study conceptualised ageing adults’ personal care expectations as shaped by their relationships with their care providers, the type of care tasks required and the availability of individual‐ and structural‐level care‐related resources. Most participants expected to care for themselves for as long as possible. Regarding the scenario in which they cannot fulfil their own needs, participants expressed preference for ageing in place with family members, followed by receiving care from community care professionals, domestic workers and other informal care sources. Institutional care was an unpopular choice because of its perceived poor care quality and was only desired in cases of extensive care needs. Findings reflected discrepancies between care preferences and reality that resulted in worries, feelings of uncertainty and ambivalent attitudes towards filial care. Unreliable family care, limited financial resources and insufficient community services caused discrepancies between ageing adults’ care preferences for various forms of informal care and the anticipated reality of poor‐quality institutional care. As caregivers, ageing adults maintained the traditional belief of children being the most desirable caregivers for their parents. However, as potential care receivers, they felt that they could not expect care from their own children for various practical reasons. Ageing adults in Hong Kong currently experience a sense of ambivalence between traditional values and current situations. The high perceived likelihood of receiving institutional care was always a result of compromise.

This study verified that older adults desire to maintain independence and self‐care before seeking help from family members (Dunér & Nordström, 2005). Studies have reported that independence and self‐care promote older adults’ self‐esteem and life satisfaction (Blair, 1999; Hertz & Anschutz, 2002). Family care preferences align with the hierarchical compensatory model (Cantor, 1991; Cantor & Brennan, 2000), which conceptualises older adults’ preferred care sources according to their degree of relation to the caregiver (Spitze & Ward, 2000). Furthermore, this study affirmed that when family care was perceived as unreliable or inadequate, paid care sources were expected and required. Institutional care was also reluctantly expected. Several participants expressed the preference of receiving personal care in familiar environments in the event of developing moderate care needs and expected institutional care only when their care needs become extensive. This finding partially supports the complimentary model in that self‐care and informal care were preferred for less burdensome care tasks, whereas formal care was preferred for more professional and extensive caregiving duties. Various care sources can complement each other and are suitable for different care tasks.

Ageing in place remained the most desirable option among the majority of participants for when they cannot care for themselves. Most participants identified family care in familiar environments as their preferred care form, although sometimes this may be unrealistic because of sociocultural changes in Chinese society. Few participants explicitly mentioned the filial obligation that is generally expected in Chinese culture (Wong & Chau, 2006), although such values were embedded in caregiving narratives. Unreliable family care appeared to preclude the influence of the cultural value of filial piety on personal care expectations. This was consistent with the findings reported by other studies that family care availability could mediate the influence of culture on older adults’ care expectations (Pinquart, Sörensen, & Song, 2018). Strong familistic beliefs did not directly predict high expectations of family care, and the accessibility and availability of family support influenced older adults’ care expectations. Therefore, instead of family care, paid personal care, such as by a domestic worker, was recognised as an alternative to fulfilling ageing adults’ care expectations. This finding is consistent with that of another study conducted in Taiwan (Chou et al., 2015). However, paid personal care remained inaccessible for many because of financial limitations.

A survey conducted by the Hong Kong Christian Service (2016) reported that approximately half of 1,096 participants older than 60 years were unsatisfied with retirement pension arrangements and the quality of nursing homes. These two concerns yielded the highest levels of disappointment among factors believed to affect older adults’ sense of hope for the future. The survey's findings echoed this study's results that along with unreliable family care and insufficient community care, financial limitations and quality of institutional care are two major barriers preventing older adults in Hong Kong from achieving desirable care arrangements. Institutional care was mainly a last resort for participants because of fears concerning poor quality of life and loss of dignity. Without effective quality control of care services, institutional care was unsurprisingly an undesirable substitute for informal care among older adults in Hong Kong. This barrier should be addressed along with the financial limitations, which drastically affect potential care options for older adults in Hong Kong.

### 4.2 Implications for practice and policy

#### 4.2.1 Self‐care through proactive planning and technological advancements

Programmes and policies should strengthen ageing adults’ proactive coping strategies to prevent or delay loss of autonomy and help them to maintain a sense of confidence in their abilities by promoting informed decision‐making and social connections for strengthening their perceived control over daily life (Matsui & Capezuti, 2008). Family members and service providers should carefully balance autonomy and care because some actions such as labelling older people as physically dependent and assisting with daily activities without evaluating their actual abilities may discourage self‐care and weaken autonomy (Sacco‐Peterson & Borell, 2004; Wikström & Emilsson, 2014). Technological advancements in assisted‐living facilities can facilitate personal care when ageing in place. Some ageing adults positively perceived modernised lifestyles, which is a finding that is consistent with that of a study conducted in China (Bai, 2016). Assistive technology devices should be developed to promote self‐care. Multidisciplinary collaboration can generate innovative solutions for meeting the care needs of ageing adults.

#### 4.2.2 Fostering ageing in place through family and community care

Changing family structures due to declining birth rates and changing living arrangements affect traditional caregiving (Bai, Liu, Baladon, & Rubio‐Valera, 2018) and ageing in place in Chinese society. Caregiver‐friendly workplace policies (e.g. flexible working arrangements, paid caregiving leave) can facilitate family care (Lai & Leonenko, 2007). Appropriate and adequate community care services also enable ageing in place regardless of personal care needs (Chui, 2008). However, home and community care services in Hong Kong are far from adequate (Fu, Chui, Kan, & Ko, 2017). Policy reforms should encourage the integration of social and technological innovations in service developments to support caregivers and facilitate ageing at home (Lynch & Draper, 2014).

#### 4.2.3 Improving institutional care to enable ageing with dignity

Although institutional care was regarded as a last resort, it was the expected outcome for many participants. Behaviour that disregards dignity can undermine older adults’ self‐esteem, self‐respect and self‐worth (Gallagher, Li, Wainwright, Jones, & Lee, 2008). Residential care should be improved by setting higher staff performance standards and intensively monitoring service delivery on the basis of residents’ health and psychosocial needs and preferences, including dignity and respect. Although participants preferred government‐subsidised care facilities, long waiting times impeded this option. As Hong Kong's ageing population grows, the average waiting time for government‐subsidised homes has become approximately 3 years (Lai, 2017). To reduce the waiting time for public institutional care of older applicants, premature institutionalisation should be reduced through a targeted policy framework that efficiently addresses residential ageing care needs and encourages community service. Moreover, the private market licensing system should be improved to ensure that private institutional care facilities comply with statutory requirements and overcome barriers to delivering quality services.

### 4.3 Limitations

This study has several limitations that should be acknowledged. First, limitations inherent to the participant recruitment strategy may have influenced sample diversity. For example, ageing adults with limited physical mobility were not included. The second limitation lies in the characteristics of the Photovoice method. The commitment required of participants to finish the entire process for the research procedure may have resulted in the participants not being representative of ageing adults in Hong Kong with respect to initiative, motivation and resilience. These limitations can be addressed by future research that adopts quantitative research methods. Large‐scale representative samples can be achieved through random sampling, and statistical analysis can be conducted to examine how personal‐, familial‐ and social‐level variables determine ageing adults’ diverse care expectations.

## 5 CONCLUSION

Guided by the compensatory and complementary models, this study revealed diverse personal care expectations among ageing adults in Hong Kong. Both current and future care recipients expressed their attitudes and perceptions regarding self‐care and informal and formal care within changing sociocultural contexts. This successful application of the photovoice research method demonstrated its value for disclosing the perspectives of ageing adults in service and policy development, which can enable their real care needs, preferences and expectations to be heard and thus addressed. Policy‐makers and service providers should facilitate self‐care and ageing in place through fostering proactive planning, technological advancement and home‐based services. Institutional care quality should also be improved to ease ageing adults’ fear of formal care and enable age in dignity.
